# Development and validation of a method for quantitative determination of the genotoxic impurity diethyl sulfate in pitolisant hydrochloride *via* high-performance liquid chromatography

**DOI:** 10.1039/d5ra01217a

**Published:** 2025-05-14

**Authors:** Santosh Bhagwat, Prakash Patil, Ramdas Pawar, Santosh Shinde, Dinesh Amalnerkar, Dnyaneshwar Shinde

**Affiliations:** a Post Graduate Department and Research Centre of Chemistry, Prof. Ramkrishna More Arts, Commerce & Science College Akurdi, Pradhikaran Pune Maharashtra India pdp1111@gmail.com sntshbhagwat@gmail.com; b Department of Chemistry, Dr Arvind B. Telang Senior College of Arts, Science and Commerce Pune Maharashtra India; c Post Graduate Department and Research Centre of Chemistry, Annasaheb Awate College Manchar Maharashtra India; d Pimpri Chinchwad University Pune 412106 India

## Abstract

A simple and highly sensitive method was devised to detect and measure minute concentrations of diethyl sulfate (DES), a potential genotoxic impurity found in the active pharmaceutical ingredients of pitolisant hydrochloride. High-performance liquid chromatography (HPLC) was employed to accurately quantify this impurity, involving pre-column derivatization with sodium phenoxide. Chromatographic separation was achieved using a Shim-pack C18 column (250 × 4.6 mm ID × 5 μ), with a mobile phase composed of 0.01 M sodium dihydrogen orthophosphate in water (mobile phase A) and acetonitrile (mobile phase B) in a gradient elution mode at a flow rate of 1.5 mL min^−1^ and the column temperature maintained at 25 °C. Detection was performed at 218 nm with an injection volume of 30 μL. Validation was conducted in accordance with standard International Council for Harmonisation of Technical Requirements for Pharmaceuticals for Human Use (ICH) guidelines, encompassing parameters such as system suitability, specificity, the limit of detection (LOD), the limit of quantification (LOQ), linearity, and accuracy. The method demonstrated an LOD and LOQ of 4 ppm and 12 ppm, respectively. This developed HPLC methodology proved to be well-suited for quantifying trace levels of the potential genotoxic impurity diethyl sulfate (DES) in pitolisant hydrochloride, a by-product originating from the synthesis process.

## Abstract 2

Diethyl sulfate, a carcinogenic by-product in pitolisant hydrochloride synthesis, is converted to ethoxybenzene using sodium phenoxide, enabling its quantification by HPLC-UV to help minimize risk in the drug substance.

## Graphical abstract

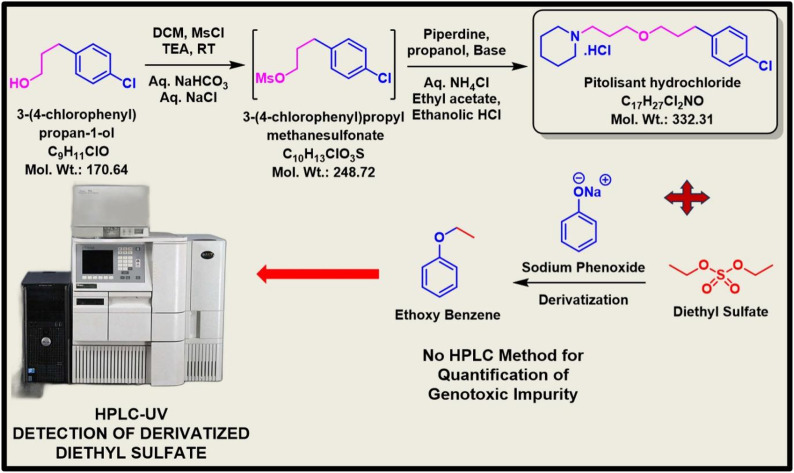

## Introduction

Pitolisant (WAKIX) functions as a selective antagonist or inverse agonist of the histamine H3 receptor, prescribed for the management of type 1 or 2 narcolepsy in adult patients.^1^ Narcolepsy, a chronic neurological condition affecting approximately 1 in 2000 individuals, is characterized by symptoms such as excessive daytime sleepiness, abnormal REM sleep patterns, sleep paralysis, and hypnagogic hallucinations.^2,3^ The chemical denomination of pitolisant hydrochloride is 1-{3-[3-(4-chlorophenyl)propoxy]propyl}piperidine hydrochloride.^4^ The production process of pitolisant hydrochloride typically involves two stages.^5^ The initial phase entails the protection of alcohol using methane sulfonyl chloride, succeeded by subsequent nucleophilic substitution with 1-piperidinepropanol.^6^ During this reaction, the by-product diethyl sulfate (DES) is formed when ethanolic hydrochloride reacts with 3-(4-chlorophenyl)propyl methanesulfonate.

Diethyl sulfate (DES) is a highly reactive compound with structural features known to be associated with genotoxicity, making it a potential genotoxic impurity (GTI), as documented in the literature.^7^ Regulatory guidelines from the European Medicines Agency (EMA), the International Council for Harmonization, and the Food and Drug Administration (FDA) stipulate the necessity for monitoring and controlling residual diethyl sulfate in pitolisant hydrochloride drug substances.^7–9^ The TTC threshold for diethyl sulfate (DES) has been determined to be 1.5 μg d^−1^. Based on the TTC limit of 1.5 μg d^−1^ and the maximum daily dose of pitolisant hydrochloride (35.6 mg), genotoxic impurities must be controlled at 40 ppm in pitolisant hydrochloride. However, no methods have been reported to determine the content of diethyl sulfate in pitolisant hydrochloride. A review of existing literature indicates a limited number of analytical methods available for assessing diethyl sulfate (DES) levels in other drug substances.

Gas chromatography (GC), particularly that coupled with headspace analysis (GC-HS), is a well-established method for detecting volatile impurities such as diethyl sulfate (DES) owing to its high sensitivity and selectivity.^10–13^ However, GC-HS techniques often require derivatization to enhance the volatility of polar impurities, which complicates the process. Additionally, the cost of consumables and instrument maintenance poses challenges for routine quality control testing. GC-MS, another advanced option, offers improved selectivity but shares similar limitations of derivatization, high operational costs, and the need for specialized training. Liquid chromatography-mass spectrometry (LC-MS) has emerged as an alternative, eliminating the derivatization^14–18^ step and offering excellent sensitivity.^19^ Despite its advantages, LC-MS systems remain inaccessible to many laboratories due to their complexity and expense. Detailed information about some impurities present in drug substances, their limits, detection techniques and associated limitations is provided in Table 1.

**Table 1:** Existing methods for the detection of diethyl sulfate (DES) and a few other genotoxic impurities, highlighting their limitations^10,17–30^

Name of impurity	Maximum daily dose	Drug substance	Limit	Detection techniques	Limitations
Dimethyl sulfate^10^	0.07 g	Methoxsalen	21.4 ppm	GC-HS	• Static headspace techniques might face limitations in reproducibly extracting volatile components like DMS from the sample matrix
Bromoethane^20^	1.0 g	Vigabatrin	1.5 ppm		• High-cost consumables required for the maintenance of GC-HS
Methyl chloride, ethyl chloride and isopropyl chloride^21^	0.160 g	Ziprasidone hydrochloride	9.4 ppm		
Dimethyl sulphate & diethyl sulphate^22^	Not applicable	General	0.05 ppm	GCMS	• Requires highly skilled analysts for setup and troubleshooting. High analysis cost, including consumables and maintenance of the mass spectrometer. The method may have longer analysis times compared to HPLC
Dimethyl sulfate^23^	0.09 g	API intermediate	16.7 ppm		
Dimethyl sulfate^24^	0.07536 g	Tipiracil hydrochloride	19.9 ppm		• Sensitive to matrix effects. Maintenance costs of both GC and MS can be high. Requires complex sample preparation and method development for accurate quantification
Alkyl camphor sulfonates^25^	Not applicable	General	1.5 ppm		
Methyl *p*-toluene sulfonate, ethyl *p*-toluene sulfonate & isopropyl *p*-toluene sulfonate^26^	4 g	Dobutamine hydrochloride	120 ppm		• Requires clean sample handling to avoid cross-contamination
Methyl-4-chlorobutyrate^27^	0.4 g	Moxifloxacin	3.75 ppm		
Allyl chloride^28^	1.2 g	Gemfibrozil	0.03 ppm		• Limited to volatile compounds—less effective for polar or non-volatile impurities
Methyl bromide (Me.-Br), ethyl bromide (Et.-Br), isopropyl bromide (Ipr.-Br), *n*-propyl bromide (*n*-Pr.-Br) and *n*-butyl bromide^29^	4.2 g	Divalproex sodium	0.5 ppm		
(4-Sulfamoylphenyl)hydrazine hydrochloride (SHH) and (4-methyl-acetophenone) *para*-sulfonamide phenylhydrazine hydrochloride (MAP)^30^	0.2 g	Celecoxib	1.0 ppm	LC-MS	• Requires careful calibration and maintenance to prevent drift. Expensive setup and consumables, especially with mass spectrometer operation
5-(4-Chlorobutyl)-1-cyclohexyl-1*H*-tetrazole^31^	0.2 g	Cilostazol	2.5 ppm		• Sensitivity can be affected by matrix effects, and detection limits for trace impurities may be impacted by column degradation over time
Hydrazine^32^	0.08 g	Pantoprazole sodium	6.25 ppm	HPLC	• Lower analysis cost compared to GC-HS. Minimal matrix effects for hydrazine. Reliable results for moderately volatile compounds like hydrazine
Benzyl chloride^33^	0.3 g	Posaconazole	51 ppm		• Simpler setup compared to GC-MS
					• HPLC is more cost-effective than LC-MS
					• Cost-effective for routine analysis

Hence due to the inherent limitation of GC-HS, GC-MS and LC-MS techniques for detecting genotoxic impurities, there are challenges in developing a simpler, cost-effective method to quantify diethyl sulfate (DES).

In this study, we propose the utilization of high-performance liquid chromatography coupled with a UV detector (HPLC-UV) to address these challenges. This method takes advantage of the UV absorption properties of derivatized diethyl sulfate (DES), optimally detected at 218 nm. The HPLC-UV method offers a balance of sensitivity, accuracy, and operational simplicity. Additionally, it provides a more economical and practical alternative to GC-MS and LC-MS for routine testing environments. A critical comparison of existing methods, including GC-HS, GC-MS, LC-MS, and the proposed HPLC-UV method for detecting genotoxic impurities, highlighting their respective advantages, limitations, sensitivity, accuracy, and operational requirements, is presented in Table 2.

**Table 2:** The comparison of existing methods and the proposed HPLC-UV method, highlighting their respective advantages, limitations, sensitivity, accuracy, and operational requirements

Parameter	GC-HS	GC-MS	LC-MS	Proposed HPLC-UV method
Sensitivity	High	Very high	Very high	High
Accuracy	High	Very high	Very high	High
Precision	Moderate	High	High	High
Cost	Moderate to high	High (expensive instrumentation)	High (expensive instrumentation)	Low (affordable instrumentation)
Sample preparation	Requires derivatization	Requires derivatization	Minimal	Minimal (direct detection)
Technical expertise	Moderate	Requires highly skilled analysts	Requires highly skilled analysts	Minimal expertise required
Operational simplicity	Moderate	Low (complex sample handling and setup)	Low (complex calibration and maintenance)	High
Matrix effects	Susceptible	Susceptible	Susceptible	Less susceptible
Detection limit	∼0.05 ppm	∼0.05 ppm	∼0.05 ppm	∼0.05 ppm
Maintenance cost	Moderate to high	Very high	Very high	Low

However, no methods have been reported to determine the content of diethyl sulfate in pitolisant hydrochloride. A review of existing literature indicates a limited number of analytical methods available for assessing diethyl sulfate (DES) levels in other drug substances.^17,18^

Nowadays, the growing need for more accessible and cost-effective methods to detect diethyl sulfate (DES) and other genotoxic impurities in drugs presents a significant challenge for researchers. In this study, we propose a simple and economical method to quantify diethyl sulfate (DES) in pitolisant hydrochloride by utilizing high-performance liquid chromatography coupled with a UV detector (HPLC-UV). HPLC-UV has the ability to detect derivatized diethyl sulfate (DES) due to its UV absorption properties, which is optimally detected at 218 nm. HPLC-UV satisfies the need for a balance between sensitivity, accuracy, and operational simplicity, unlike more expensive and technically demanding methods such as GC-MS and LC-MS. Validation of the method proved its ability to deliver reproducible results in routine testing environments, with recovery rates demonstrating its precision.

The approach taken by this study is innovative in addressing the need for a practical, cost-effective, and accessible method to monitor diethyl sulfate (DES) in pitolisant hydrochloride drug substances. In conclusion, this research introduces a viable solution for routine diethyl sulfate (DES) detection in pitolisant hydrochloride, providing a practical analytical tool that can be widely adopted to ensure patient safety without incurring the high costs associated with GC-MS or LC-MS technologies.

## Materials and methods

### Chemicals and reagents

The experimental chemicals, including diethyl sulfate (DES) (>99%), phenol (>99%), sodium hydroxide, sodium dihydrogen phosphate, and acetonitrile of HPLC grade, were obtained from Merck Life Science Pvt. Ltd, Pune, India, and used without additional purification or processing. De-ionized water was generated through an in-house Milli-Q water purification system (Millipore, Billerica, MA). Pitolisant hydrochloride samples (99.5%) targeted for the assessment of genotoxic impurity content were sourced from Bhisaj Pharma Pvt. Ltd, Pune, India.

### Equipment

The method development to determine the diethyl sulfate (DES) content in pitolisant hydrochloride involved the utilization of a Shimadzu HPLC system featuring a G4226A 1290 autosampler, a G4220A 1290 binary pump, a G1316C 1290 thermostatic column oven, and a PDA detector with a G4212A 1290 photodiode array detector. The chromatographic separation was conducted using a Shim-pack C18 column (5 μm, 4.6 × 250 mm). Additionally, a Mettler Toledo XP 205 microbalance from Columbus, Ohio, and an 8510 Branson Sonicator from Danbury, CT, were employed for weighing and sonication procedures, respectively.

### Derivatization reaction

For method development, a solution containing 1000 ppm of diethyl sulfate (DES) in a 1 : 1 mixture of acetonitrile and water was prepared and subjected to analysis utilizing an HPLC-UV detector under various conditions presented in Table 3. However, the chromatogram did not exhibit any distinct peaks, indicating that diethyl sulfate (DES) does not possess a chromophore, rendering it undetectable by the HPLC-UV detector. To address this limitation, a strategy was devised to introduce a chromophore capable of facilitating detection by the HPLC-UV detector. Consequently, several derivatization reactions were explored, including benzaldehyde, benzyl chloride, 2-nitrophenol, and sodium phenoxide. Following multiple derivatization experiments, sodium phenoxide was found to be the most suitable derivatizing reagent due to its greater nucleophilicity and faster reaction rate with diethyl sulfate (DES), forming ethoxybenzene. Hence, we used NaOH for the preparation of sodium phenoxide, and it was adopted for further analysis.

**Table 3:** Summary of derivatizing reagents and observations

Trial no.	Derivatizing reagent	Observations
1	Without derivatizing reagent	No DES peak observed
2	Benzaldehyde	No derivative peak observed
3	Benzyl chloride	No derivative peak observed
4	2-Nitrophenol	No derivative peak observed
5	Sodium phenoxide	Derivative peak observed

## Derivatization reaction

(1) Preparation of sodium phenoxide:
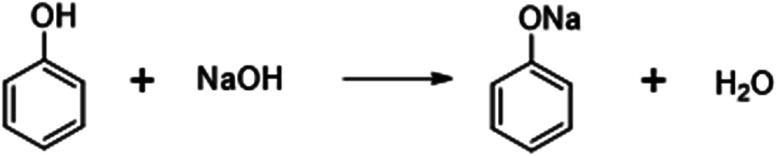


(2) Preparation of ethoxy benzene:
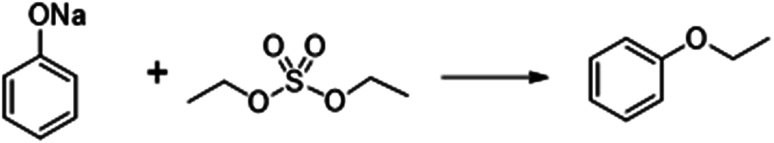


For the conversion of diethyl sulfate (DES) into ethoxybenzene, a freshly prepared solution comprising phenol and sodium hydroxide (10.5 g of phenol and 4 g of sodium hydroxide dissolved in 250 mL of water) was used, along with acetonitrile (250 mL) as the solvent. To prepare the sample solution, 100 mg of the drug substance was introduced into a 10 mL volumetric flask and then diluted with the solvent. The flask was sealed using a glass stopper, subjected to sonication, and vigorously agitated until the derivatization process reached completion. Following this, aliquots of the samples were directly introduced into the HPLC system. Multiple experimental trials were conducted to evaluate potential interference from blank samples with derivative peaks and to ensure the specificity of other intermediates.

### Standard and sample solution preparations

#### Preparation of phenol and sodium hydroxide solution

10.5 g of phenol and 4 g of sodium hydroxide were accurately weighed and transferred into a 500 mL volumetric flask. Subsequently, approximately 250 mL of water was added to dissolve the phenol and sodium hydroxide. The volume was then adjusted to the mark using acetonitrile.

#### Preparation of stock solution of diethyl sulfate (DES)

40 mg of diethyl sulfate (DES) was precisely weighed and transferred into a 100 mL volumetric flask, where it was dissolved in a solution of phenol and sodium hydroxide. The volume was adjusted to 100 mL.

#### Preparation of standard solution of diethyl sulfate (DES)

1.0 mL of the diethyl sulfate (DES) stock solution was transferred into a 100 mL volumetric flask and adjusted to 100 mL using a solution of phenol and sodium hydroxide. Subsequently, 5.0 mL of the aforementioned solution was pipetted into a 50 mL volumetric flask, and the volume was adjusted with the phenol and sodium hydroxide solution.

#### Preparation of test solution of pitolisant hydrochloride

The test sample (100 mg) was accurately weighed in a 10 mL volumetric flask, followed by dissolving and diluting it to 10 mL using a solution of phenol and sodium hydroxide.

## Results and discussion

### Chromatographic conditions

The process for determining diethyl sulfate involves three main stages: derivatization of diethyl sulfate into a product containing a chromophore, selection of chromatographic conditions, and calculation to quantify diethyl sulfate in the test sample. To efficiently analyze the derivatized product, a reversed-phase LC method was created. Ascentis Express C18, Waters Symmetry C18, Zorbax Eclipse XDB-C18, and Shim-pack C18 were among the columns with various stationary-phase chemistries that were examined during the method development process. As stated earlier, the optimal chromatographic conditions involves the early elution of the components of the API matrix, away from the diethyl sulphate (DES) derivative. The Shim-pack C18 column was chosen after considerable deliberation because of its excellent retention for the diethyl sulfate (DES) product that has been derivatized to ethoxybenzene. Furthermore, the Shim-pack C18 column was chosen over other C18 columns because of its more affordable price and stable baseline. A gradient mobile phase was used to elute the column: mobile phase A (MPA) was 0.01 M sodium dihydrogen orthophosphate in water; and mobile phase B (MPB) was acetonitrile. The quantification of diethyl sulfate is performed using eqn (3). Table 4 provides an overview of the HPLC method parameters.

Summary of the final HPLC method

**Table:** 

Parameters	Conditions
HPLC column	Shim-pack C18, 5 μm, 4.6 × 250 mm
Mobile phase	A: 0.01 M sodium dihydrogen phosphate in water, B: acetonitrile
Injection volume	30 μL
Column temperature	25 °C
Flow	1.5 mL min^−1^
Wavelength	218 nm

**Table:** 

Parameters	Conditions		
	Time (min)	% Eluent A	% Eluent B
Gradient	0	60	40
	2	60	40
	15	35	65
	25	35	65
	25.1	60	40
	30	60	40

**Table:** 

Parameters	Conditions
Diluent	10.5 g of phenol and 4 g of sodium hydroxide to 250 mL of water, and added 250 mL of acetonitrile to this solution
Standard preparation	0.4 ppm in diluent
Sample preparation	10 000 ppm in diluent

**Fig. 1:**
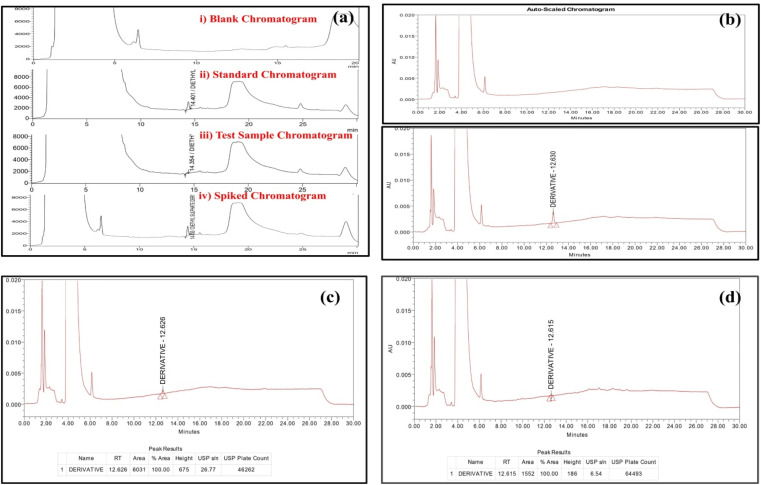
(a) Comparison of the blank, standard, test, and spike chromatograms. (b) Chromatograms of ethoxybenzene standard and blank. (c) Ethoxybenzene standard peaks at the LOQ (12 ppm in 10 mg per mL API). (d) Ethoxybenzene standard peaks at the LOD (4 ppm). Chromatographic conditions: Shimadzu HPLC system with UV detection at 218 nm; column: Shim-pack C18 (250 mm × 4.6 mm, 5 μm); mobile phase A: 0.01 M sodium dihydrogen phosphate in water; mobile phase B: acetonitrile, flow rate: 1.5 mL min^−1^; injection volume: 30 μL; column oven temperature: 25 °C.

Fig. 1 shows the chromatograms of diethyl sulfate derivative under optimized HPLC conditions. Fig. 1a depicts the comparison of chromatograms of blank, standard, test, and spiked samples, showing peak shape and resolution. Fig. 1b shows the chromatogram of blank and diethyl sulfate (DES) derivative (ethoxybenzene) standard. Fig. 1c shows the limit of quantification peak (LOQ, 12 ppm in 10 mg per mL API) of the diethyl sulfate derivative ethoxybenzene standard. Fig. 1d shows the limit of detection (LOD, 4 ppm in 10 mg per mL API) of the diethyl sulfate derivative ethoxybenzene standard.^19^

### Calculation for standard concentration



1

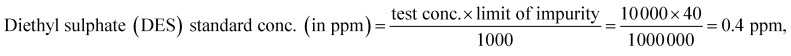

where

2 40: limit of impurity in ppm = 40 μg L^−1^ = 0.04 mg mL^−1^ (ref. **27**)

### Calculation of diethyl sulfate (DES)



3

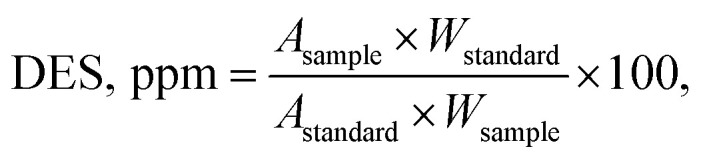

where *A*_sample_ = peak area of diethyl sulfate derivative in the test solution chromatogram. *A*_standard_ = peak area of diethyl sulfate derivative in the standard solution chromatogram. *W*_standard_ = weight of diethyl sulfate for the standard solution, mg. *W*_sample_ = weight of sample in the test solution, mg. 100 = dilution factor for typical preparations.4
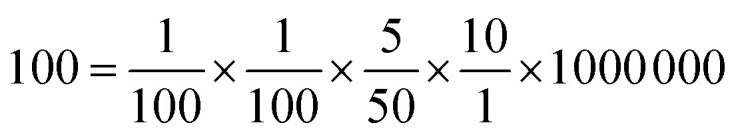


### Method validation

The method underwent validation following the guidelines outlined in ICH Q2(R1) to assess parameters including specificity, limit of detection (LOD) and limit of quantification (LOQ), linearity and range, accuracy, and precision, ensuring its suitability for the intended application.

### Specificity

Diethyl sulfate (DES) and pitolisant hydrochloride solutions were injected separately to show specificity.^5,20,21^ At the diethyl sulfate (DES) retention time, no interference peaks were seen in the blank chromatogram (Fig. 1).^34–38^

### Limit of detection & limit of quantification

The limit of detection (LOD) and limit of quantification (LOQ) were established based on the signal-to-noise (S/N) approach, in accordance with ICH Q2(R1) guidelines. According to this approach, the LOD corresponds to a signal-to-noise ratio of approximately 3 : 1, while the LOQ corresponds to a signal-to-noise ratio of approximately 10 : 1. Using this method, both parameters were determined from the diethyl sulfate (DES) stock solution. Based on the obtained chromatographic responses, the LOD was found to be 4 ppm and the LOQ was determined to be 12 ppm. At the LOQ level, the mean S/N ratio was 26, and the relative standard deviation (RSD) of the peak area was 5.22%, demonstrating acceptable precision. Alternatively, these values can be calculated using specific formulas: LOD = 3.3 × *σ*/*S* and LOQ = 10 × *σ*/*S*, where *S* denotes the slope of the line equation and *σ* represents the standard deviation of the *y*-intercept of the line^39–43^ (Table 5, 6 and Fig. 1).

**Table 5:** Limit of detection of diethyl sulfate

Sr. no.	Area	S/N
1	1552	7
2	2115	7
3	1251	6

**Table 6:** Limit of quantification of diethyl sulfate

Sr. no.	Area	S/N
1	6031	27
2	5819	26
3	5693	27
4	5273	24
5	5712	24
6	5315	25
Mean	5640.500	26
SD	294.36	—
% RSD	5.22	—

### Linearity

**Fig. 2:**
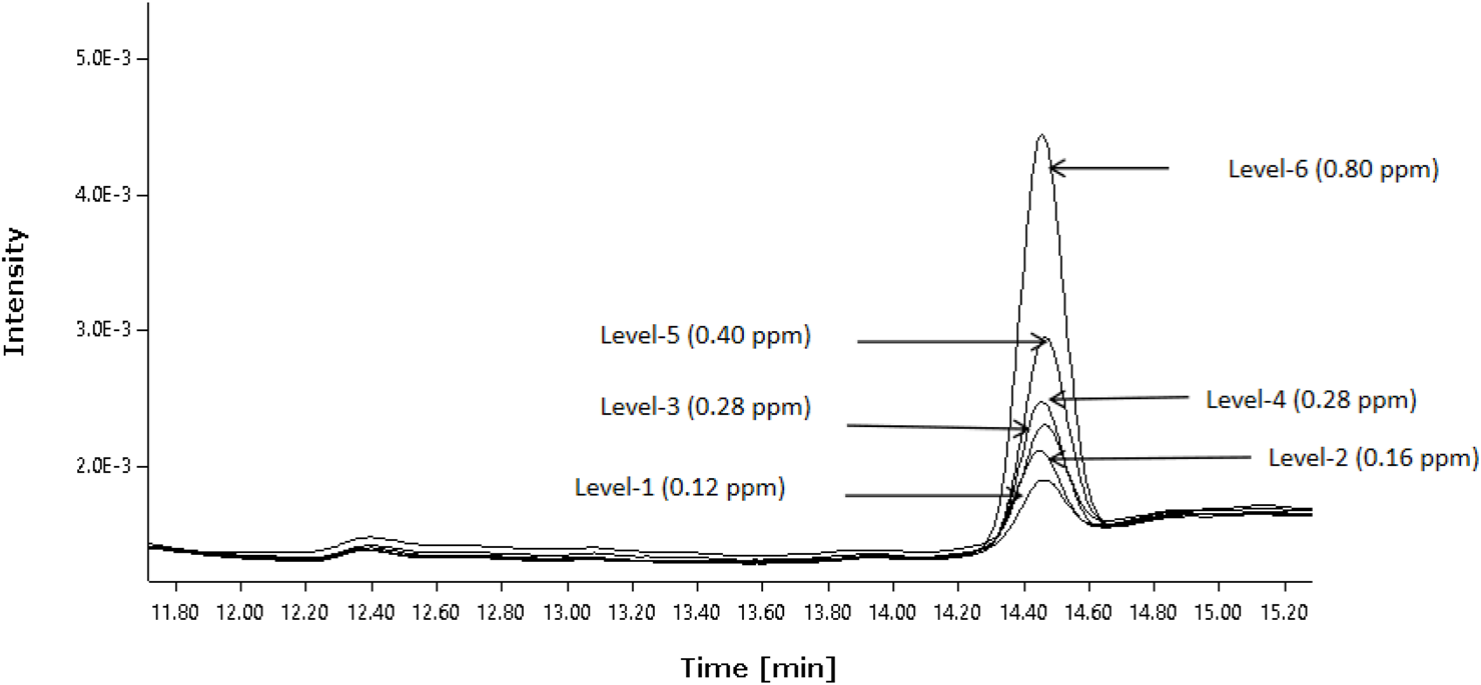
Overlay chromatograms of linearity levels.

The linearity of the present method was evaluated by injecting a sequence of diethyl sulfate (DES) concentrations spanning from the LOQ level up to 200% level. The correlation coefficient exceeded 0.99, satisfying the validation criteria^25,26^ (Table 7 and Fig. 2).

**Table 7:** Linearity of diethyl sulfate

Linearity level, %	Concentration in ppm	Area of diethyl sulphate	Response factor
(LOQ) 30	0.12	4041	33 549
40	0.16	6383	39 745
50	0.20	7688	38 296
70	0.28	9947	35 392
100	0.40	14 534	36 199
200	0.80	29 668	36 946
**Slope**	**36 864**	**Mean value of response factor**	**36 688.0448**
***Y*-intercept**	**−43.954**	**Standard deviation**	**2180.2322**
**Correlation coefficient**	**0.9984**	**% Relative standard deviation**	**5.94**

### Precision

The precision of an analytical method denotes the level of concordance among a set of measurements acquired from multiple samplings of a uniform sample under defined conditions. Precision is commonly evaluated through studies conducted on homogeneous samples. In this study, the relative standard deviation (% RSD) for the area observed from six preparations of the sample solution was 7.87%^27^ (Table 8).

**Table 8:** Method precision of diethyl sulfate

Sr. no.	Area
1	1344
2	1073
3	1118
4	1200
5	1174
6	1218
Mean	1188
STD	93.4889
% RSD	7.87

### Accuracy

The accuracy of an analytical method denotes its capacity to furnish results close to the actual value. To validate the method's recovery, predetermined quantities of diethyl sulfate (DES) were added to pitolisant hydrochloride test specimens at four distinct levels: the limit of quantification (LOQ), 50%, 100%, and 150% of the 0.4 ppm standard concentration. The accuracy results ranged from 75% to 106% across all levels (Table 9).

**Table 9:** Accuracy results of diethyl sulfate

Accuracy	Level-I	Level-II	Level-III	Level-IV
Amount added	11.9641	19.8847	39.7575	59.8802
Amount found	9.0441	20.9769	38.2274	56.4604
% Recovery	75.6%	105.5%	96.2%	94.3%

## Conclusion

A simple derivatization reaction and a universal HPLC-UV detector were employed to develop a simple and sensitive method for quantifying diethyl sulfate (DES) in pitolisant hydrochloride. Validation tests conducted on the method indicate its precision, sensitivity, linearity, and accuracy. Thus, this method presents a convenient approach for determining diethyl sulfate (DES) levels in pitolisant hydrochloride test samples. We assume that the proposed methodology for determining the diethyl sulfate (DES) impurity in pitolisant hydrochloride drug substances will be cost-effective, easy to handle, and time-efficient.^44–46^

## Data availability

All data generated or analyzed during this study are included in this published article.

## Conflicts of interest

The authors declare no conflicts of interest associated with this study.
